# Development of a trash classification system to map potential *Aedes aegypti* breeding grounds using unmanned aerial vehicle imaging

**DOI:** 10.1007/s11356-024-33801-0

**Published:** 2024-06-06

**Authors:** Joelle I. Rosser, Morgan S. Tarpenning, Juliet T. Bramante, Anoushka Tamhane, Andrew J. Chamberlin, Paul S. Mutuku, Giulio A. De Leo, Bryson Ndenga, Francis Mutuku, Angelle Desiree LaBeaud

**Affiliations:** 1 School of Medicine, Division of Infectious Diseases, Stanford University, Stanford, CA USA; 2 Stanford University, Stanford, CA USA; 3 School of Medicine, University of Washington, Seattle, WA USA; 4 BASIS Independent Silicon Valley, Palo Alto, CA USA; 5 Hopkins Marine Institute, Department of Earth System Sciences and Department of Oceans, Stanford University, Stanford, CA USA; 6 Division of Vector Borne Disease Control Unit, Msambweni County Referral Hospital, Msambweni, Kenya; 7 Centre for Global Health Research, Kenya Medical Research Institute, Kisumu, Kenya; 8 Department of Environment and Health Sciences, Technical University of Mombasa, Mombasa, Kenya; 9 School of Medicine, Division of Pediatric Infectious Diseases, Stanford University, Stanford, CA USA

**Keywords:** *Aedes*, Garbage, Trash, Waste, Unmanned aerial device, Unmanned aerial vehicle, Remote sensing technology, Vector borne diseases

## Abstract

*Aedes aegypti* mosquitos are the primary vector for dengue, chikungunya, and Zika viruses and tend to breed in small containers of water, with a propensity to breed in small piles of trash and abandoned tires. This study piloted the use of aerial imaging to map and classify potential *Ae. aegypti* breeding sites with a specific focus on trash, including discarded tires. Aerial images of coastal and inland sites in Kenya were obtained using an unmanned aerial vehicle. Aerial images were reviewed for identification of trash and suspected trash mimics, followed by extensive community walk-throughs to identify trash types and mimics by description and ground photography. An expert panel reviewed aerial images and ground photos to develop a classification scheme and evaluate the advantages and disadvantages of aerial imaging versus walk-through trash mapping. A trash classification scheme was created based on trash density, surface area, potential for frequent disturbance, and overall likelihood of being a productive *Ae. aegypti* breeding site. Aerial imaging offers a novel strategy to characterize, map, and quantify trash at risk of promoting *Ae. aegypti* proliferation, generating opportunities for further research on trash associations with disease and trash interventions.

## Background

*Aedes aegypti* mosquitoes, which can be found globally in tropical and sub-tropical climates, are the primary vector for multiple arboviruses including dengue, chikungunya, and Zika viruses (Kraemer et al. 2015). *Ae. aegypti* is a highly anthropophilic species that commonly breeds in small, man-made containers of water such as plastic containers, bottles, buckets, and other trash that can collect rainwater (Getachew et al. 2015; Ngugi et al. 2017, 2020; Krystosik et al. 2020; Forsyth et al. 2020, 2022; Nosrat et al. 2021; Mwakutwaa et al. 2023; Khan et al. 2023). Discarded tires are uniquely suited to holding rainwater and serve as a particularly productive breeding ground (Hayes et al. 2003; Sekhon and Minhas 2014). Despite trash, including tires, being a well-known breeding site for *Ae. aegypti* mosquitoes, data is limited evaluating the relationship between living in close proximity to trash and the risk for *Ae. aegypti*-transmitted infectious diseases (Khan et al. 2023; Peña-García et al. 2023). This gap is largely due to a lack of precise tools to quantify and map trash distribution. Trash has previously been characterized by walking through communities and documenting the presence of potential mosquito breeding ground (Heukelbach et al. 2001) or counting specific trash types within and directly surrounding a household (Hayes et al. 2003; Brunkard et al. 2007; Kenneson et al. 2017; Mukhtar et al. 2018). Trash exposure has also been evaluated by household distance from public landfills (Tomita et al. 2020), as well as with interviews asking about household trash contact (Zolnikov et al. 2023). However, these methods are time consuming and labor intensive, do not necessarily account for trash disposal practices or exposure at both a household and neighborhood scale, and do not provide a quantifiable measure of trash exposure.

A novel approach to surveying trash and mosquito habitats is with aerial imaging using unmanned aerial vehicles (UAVs). UAV imaging is increasingly being used to map high risk habitats for various mosquito species that transmit human pathogens like malaria and dengue (Landau and Van Leeuwen 2012; Hardy et al. 2017; Carrasco-Escobar et al. 2019; Sarira et al. 2020; Case et al. 2020; Schenkel et al. 2020; Valdez-Delgado et al. 2021; Lee et al. 2021). UAV imaging to identify *Ae. aegypti* habitat has been piloted in Ecuador (Lee et al. 2021), Mexico (Valdez-Delgado et al. 2021), and the USA (Schenkel et al. 2020), and is being used for related applications such as mapping the habitat of different mosquito species (Hardy et al. 2017; Carrasco-Escobar et al. 2019; Case et al. 2020) and assisting with beach trash cleanups (Andriolo et al. 2021; Liao and Juang 2022). Moreover, the image data generated with UAVs are well suited for automatic image detection using tailored machine learning algorithms, a strategy that has been implemented with varying success for quantifying individual containers (Case et al. 2020; Passos et al. 2021; Liao and Juang 2022), marine and beached trash (Andriolo et al. 2021, 2022), as well as land cover that provides mosquito habitat (Carrasco-Escobar et al. 2019; Trujillano et al. 2023). However, there is currently a lack of research applying these technologies to identify trash piles that pose a risk of serving as *Ae. aegypti* breeding sites.

Not all trash translates into a potential risk for *Ae. aegypti* breeding. Trash that is removed frequently or disturbed often by cars or foot traffic, for example, may not hold water consistently and therefore does not provide suitable breeding ground for mosquitoes. Trash dump sites also have a variety of appearances, both on aerial imaging and as seen when walking through a community. The lack of a trash classification system based on trash appearance and *Ae. aegypti* risk limits our ability to use UAV imaging to identify and quantify trash, and ultimately assign trash scores to particular geographic areas for further evaluation of the relationship between trash and potential risk of *Ae. aegypti* breeding. The objective of this study is to develop a trash classification system that can be applied to UAV aerial imaging to assign trash categories and risk for *Ae. aegypti* breeding habitats.

## Methods

### Study site

**Fig. 1:**
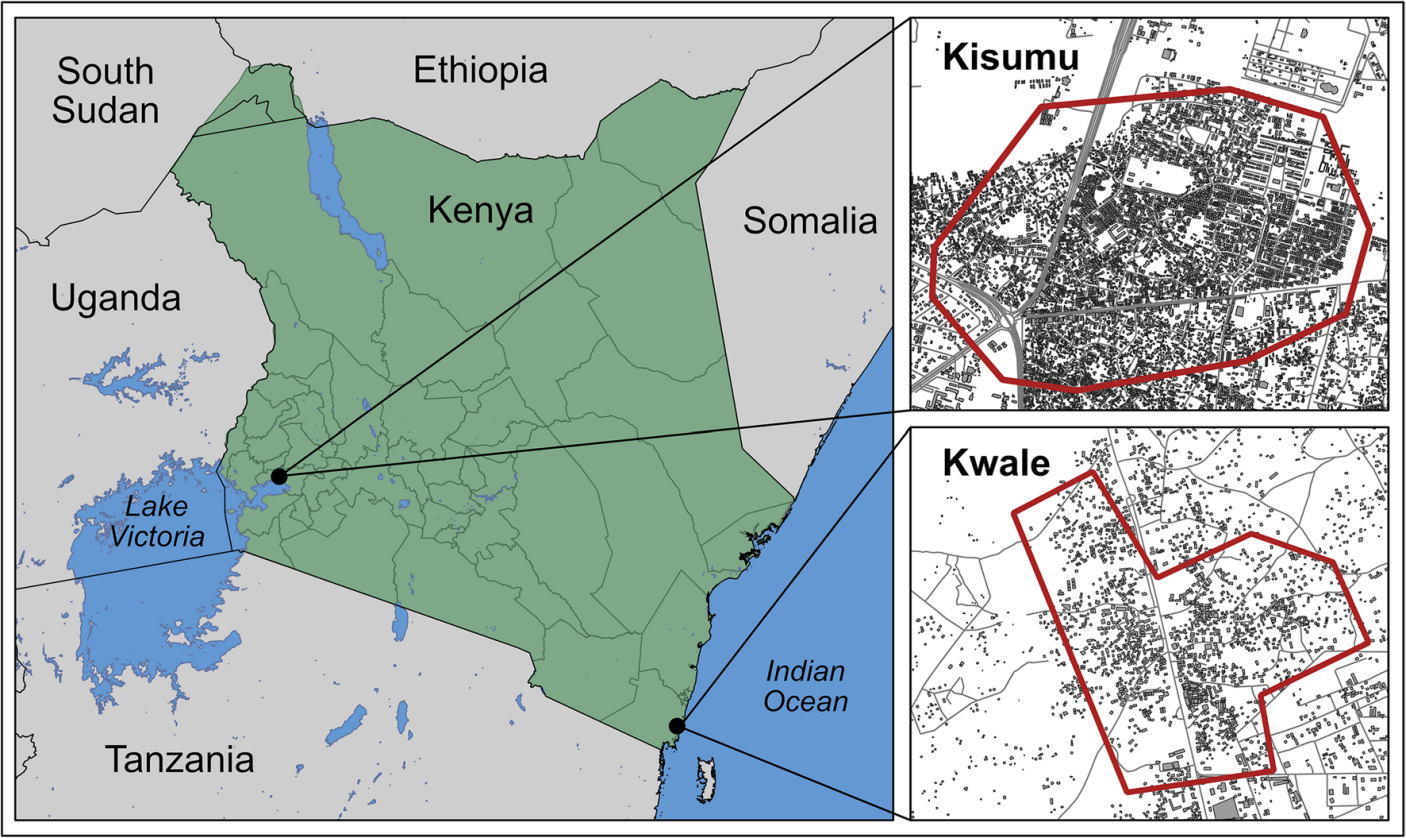
Study sites. Our study sites included the more urban, inland region of Kisumu, and the coastal, semi-rural area of Kwale in Kenya. Drone flight areas are outlined in red. County administrative boundaries available from OCHA Regional Office for Southern and Eastern Africa (https://data.humdata.org/dataset/cod-ab-ken). Country boundaries provided by IGAD Climate Prediction and Applications Centre (ICPAC) (https://geoportal.icpac.net/layers/data0:geonode:afr_g2014_2013_0). Hydrologic regions provided by ArcGIS Living Atlas Team (https://hub.arcgis.com/datasets/arcgis-content::world-lakes/about). Building and road data available from Humanitarian OpenStreetMap Team (https://data.humdata.org/dataset/hotosm_ken_buildings, https://data.humdata.org/dataset/hotosm_ken_roads)

This study was conducted in Kwale on the coast of Kenya, and Kisumu in southwestern Kenya (Figure 1). These sites are known to have extensive *Ae. aegypti* mosquitoes breeding in trash and discarded tires throughout the communities and consequent dengue and chikungunya infections (Ngugi et al. 2017).

### UAV flight planning and image acquisition

All flights plans were created using DJIFlightPlanner software version 2.5.1.15 (https://www.dji.com/mobile/downloads/djiapp/dji-pilot). Fights were conducted using a DJI Mavic 2 UAV by a licensed UAV pilot in collaboration with SwiftLabs (https://swiftlab.tech/). All flights adhered to the Kenyan Aviation Authority regulations for UAVs. Images were obtained at an altitude of approximately 100 meters over 8 days in July 2022 and January 2023 during daytime hours. Flight launch times were determined to optimize UAV flight conditions, minimizing shadows and glare from the white sand. Data was collected at two points of the year in order to capture variations in the appearance of trash across seasons.

### Aerial image processing

Image processing was done using AgiSoft Metashape Professional version 1.8.4 and base maps were generated in geographic coordinate system WGS 84 with a pixel resolution of 0.03 square meters and exported as a geotiff. Image classification and the coupling of ground truth photos with their corresponding UAV map locations were conducted in QGIS version 3.24. Aerial maps of the two study sites were systematically reviewed by two individuals to identify suspected trash and trash mimics to help direct the community walk-through evaluations.

### Walk-through identification of trash versus mimics

After reviewing the initial aerial maps, over 10 hours and 768 km^2^ of community walk-throughs were conducted with a community liaison and a community leader, both familiar with the major community trash sites and overall environment. Walk-throughs in both study sites were performed over 7 days in January 2023, concurrent with the UAV flights. During the walk-throughs, various types of trash piles, tires, and other piles that could be mistaken for trash were discussed. Photographs of various trash types and trash mimics were taken to facilitate future discussion as a group.

### Trash classification

Based on a representative sample of aerial images and ground truth photo images, trash types were iteratively classified by a team of six analysts, including two local entomologists with extensive experience evaluating *Ae. aegypti* breeding habitats in these areas. A trash classification scheme was developed based on the appearance and volume of the trash; then each trash category was assigned a risk score of high, medium, or low based on the likelihood that trash type could be a productive *Ae. aegypti* breeding site. A few types of trash were determined to be essentially no risk. Trash determined to be “no risk” and trash mimics were subsequently excluded from aerial classification. In addition to creating major trash categories, four sub-categories were created based on discussion about other environmental features around the trash that could modulate *Ae. aegypti* breeding site risk. With this structure, each trash pile can be assigned one major trash classification in addition to up to four subcategories.

### Ethics

Ethical approval for this study was obtained from the Technical University of Mombasa (TUM), National Commission for Science, Technology, and Innovation (NACOSTI), and Stanford University. Local administrative approvals were also obtained starting from the County Commissioners of Kisumu and Kwale counties down to Assistant Chiefs. Due to COVID-19 restrictions, local public meetings (*barazas*) were not conducted.

## Results

### Trash identification and classification

In the coastal site of Kwale, 1.5 km^2^ were mapped by UAV with 1316 trash piles identified. In the inland site of Kisumu, 2.0 km^2^ were mapped with 1888 trash piles identified. A total of 961 photos of trash and trash mimics were taken and reviewed by at least two team members and compiled for targeted review by the full expert panel. Trash areas, including discarded tires, were first categorized based on appearance and expert opinion of overall risk of being an *Ae. aegypti* breeding site on an ordinal scale, then could be assigned up to four sub-categories that modulated risk (Table 1).

**Table 1:** Trash classification by *Aedes aegypti* breeding habitat risk

**Trash categories**	***Aedes aegypti*** **risk**	**Trash density**	**Trash area**	**Rainwater holding capacity**	**Disturbance**
Trash collection center	High (6)	High	Large	High	Low
Large community dump	High (5)	High	Large	Medium	Low
Medium community dump	Medium (4)	High	Medium	Medium	Medium
Small household trash pile	Medium (3)	Medium	Small	Small	Medium
Scattered trash in the grass	Low (2)	Low	Large	Small	Medium
Trash pile next to water canal	Low (1)	Medium	Small	Small	High
Scattered trash by the road	-	-	-	None (Trampled)	High
Trash inside a water canal	-	-	-	High (dirty; submerged)	-
**Tire categories**					
Discarded car tire	High	NA	Small	High	Low
Tire embedded in the ground	-	-	-	None (cannot hold water)	-
**Trash sub-categories**		**Obscures view**	**Provides shade**	**High turn over**	**More likely to change**
Mixed with vegetation	Increased	Yes	Yes	-	-
Partially hidden by trees	Increased	Yes	Yes	-	-
Evidence of burning	Decreased	-	-	Yes	-
Inside building construction site	-	-	-	-	Yes

Trash piles were first classified by a primary risk category that took into account trash density, surface area, capacity to hold rainwater, and likelihood of site being disturbed. Tires were similarly classified into two categories based on the ability to hold water. Trash and tires could then be further assigned up to four sub-categories which could affect aerial visualization or breeding site risk

### Trash major risk categories

**Fig. 2:**
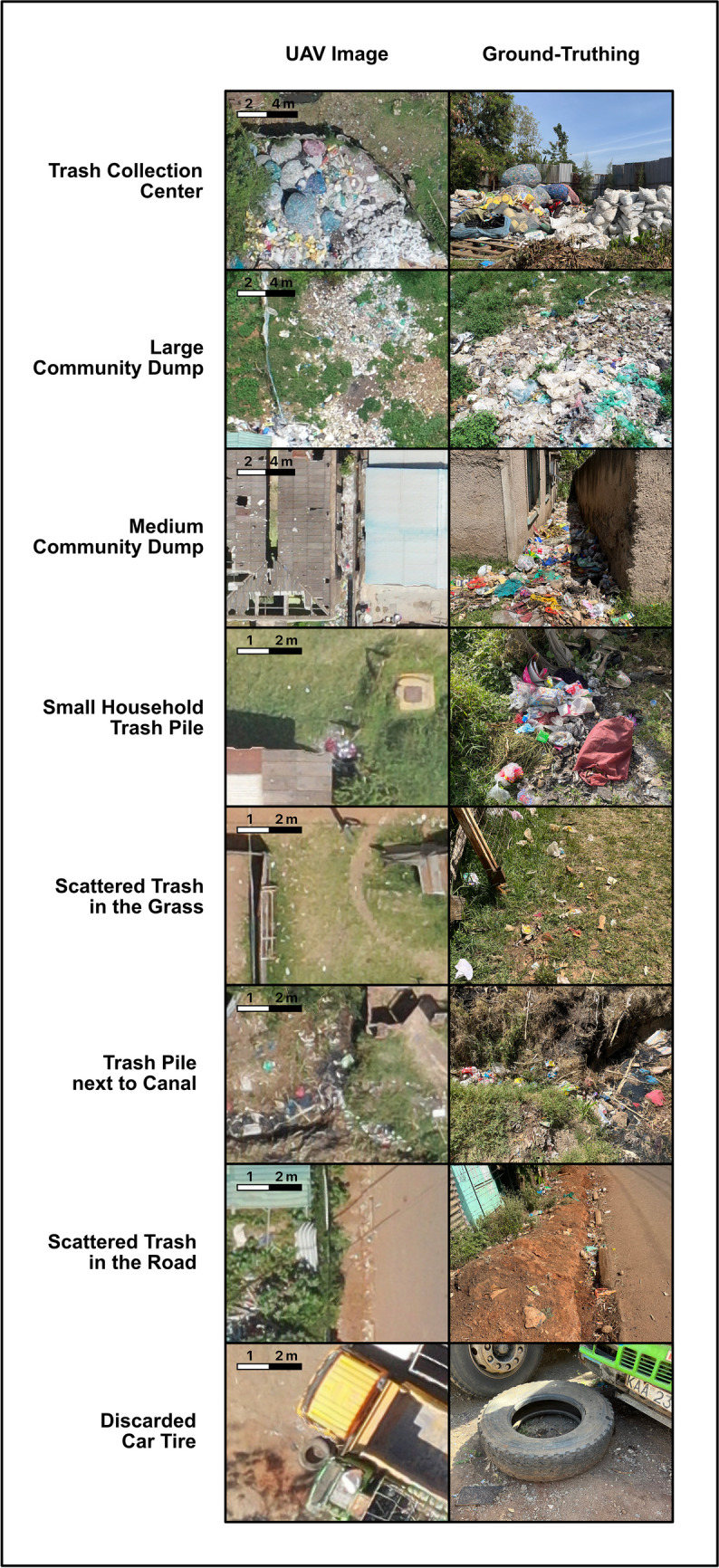
Major categories. The trash classification scheme was iteratively developed based on aerial image and ground truth visualization. Side by side comparisons are shown of the aerial image and ground truth image of major categories

The highest risk trash areas identified were trash collection centers and large community dumps (Figure 2). Collection centers typically sort trash based on material type; turnover of collected trash ranges from weeks to months, depending on the material type and time required to collect a volume that is cost-effective to transport to a larger recycling center. These large, stable piles of trash collect water which remain largely undisturbed and are ideal breeding sites for *Ae. aegypti*. Large community dumps are unsorted trash sites where multiple households and businesses dump organic and non-organic waste, typically including a large variety of small plastic containers which can breed *Ae. aegypti*; these sites are not routinely removed and experience minimal disturbance. Medium community dumps are similar to large community dumps in composition and disruption but generally smaller in area and density. Small household trash piles are where one or a small number of households dump trash; density and area of small piles is less than medium dumps and they are typically in areas around roads and households that would be expected to get more frequent foot traffic and consequent disturbance.

Scattered trash and trash within canals were divided into risk and no risk categories based on location. Scattered trash in a grassy area often consists of plastic bottles or bags that can fill with water and are relatively undisturbed, characteristics of good breeding grounds. However, because less trash is scattered over a larger area, the surface area overestimates risk compared to denser dump sites. Trash scattered in the road is frequently disturbed or trampled and therefore even plastic containers in these sites do not serve as good breeding sites and are considered no risk. A pile of trash that accumulates adjacent to a canal has some potential to fill with clean rainwater and be a low risk *Ae. aegypti* site. However, trash inside water canals is typically submerged in large amounts of dirty water which are good habitats for several *Culex* species but not *Ae. aegypti*. Therefore, trash within a canal was classified as no risk.

Discarded tires were classified based on positioning. Discarded car tires lying on the ground or leaning against something can fill with water and are a high-risk site for *Ae. aegypti*. However, tires embedded in the ground, often serving as seating areas or barriers along the roadside, cannot fill with rainwater and are no risk. The positioning and therefore risk of the tire can be differentiated on aerial imaging by the shape.

### Trash sub-categories

**Fig. 3:**
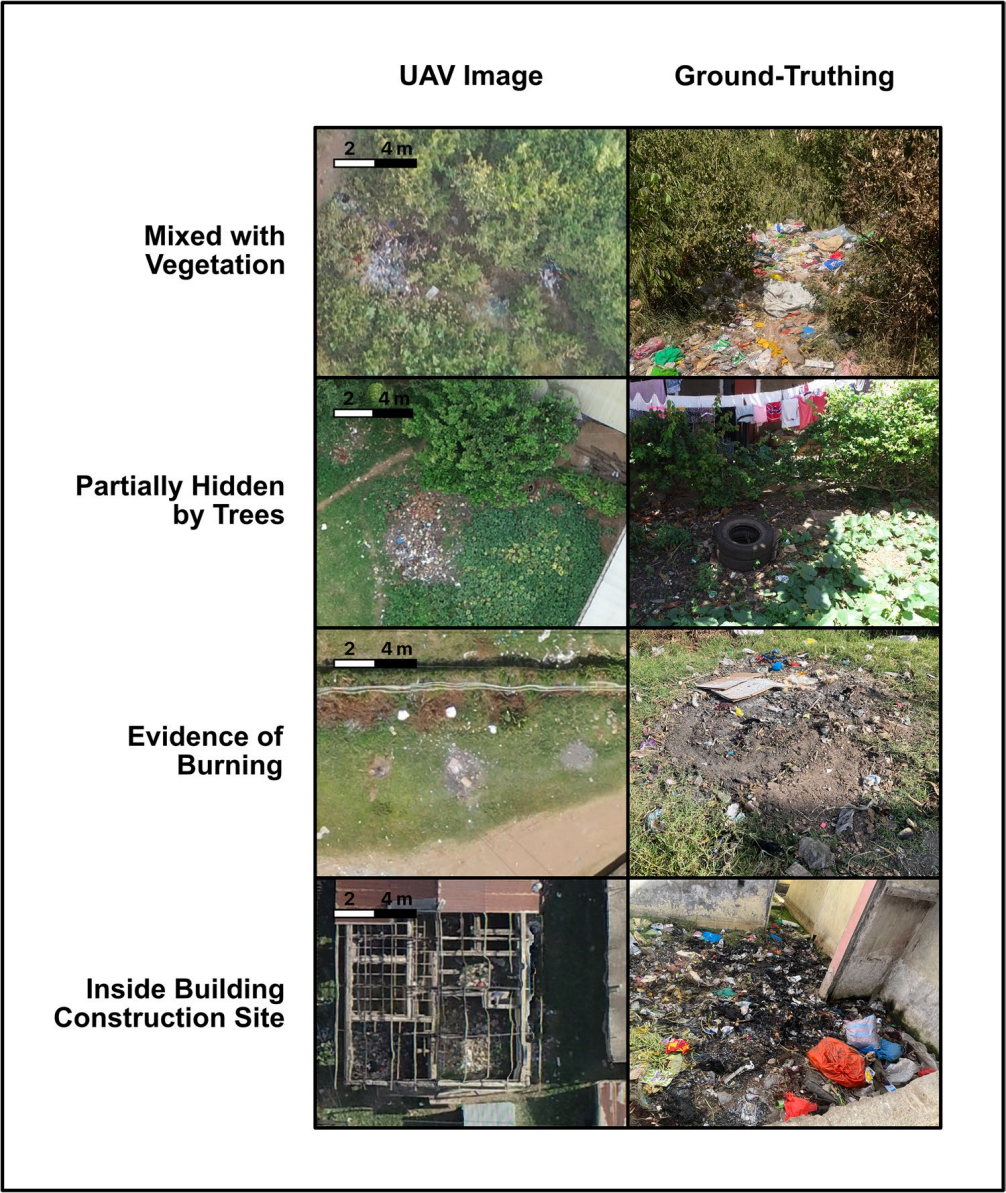
Trash classification sub-categories. Each trash pile could also be assigned to a sub-category. Side by side comparisons are shown of the aerial image and ground truth image of each sub-category

In addition to major trash categories, trash can belong to up to four sub-categories which can attenuate the *Ae. aegypti* risk, obscure the full view of trash, or be more likely to change in the near future (Figure 3). Vegetation and trees can provide shade, slowing the rate of water evaporation from small containers of water, helping breeding sites persist long enough for larval maturation. These areas can also provide nectar for adult mosquitoes and shade to protect them from desiccation. However, vegetation and trees can also obscure the aerial view of relatively large dump sites, underestimating the full extent of the site. Burning trash is a common practice, particularly for smaller piles of household trash; burning removes much of the trash and indicates a site that is frequently disturbed which decreases risk of *Ae. aegypti* breeding. Some entirely charred areas with no surrounding trash are also evident in communities and indicate areas of previous trash burning or burning for other uses such as outdoor cooking; these entirely burnt areas are no risk and are not included in the trash classification. Trash is frequently dumped in partially completed building sites as evidenced by an outline of bricks. Since these building may be undergoing active construction, these sites may become covered up or removed as construction continues; these dumps may therefore change on a shorter time horizon than other sites and pose more transient risk.

### Trash mimics

**Fig. 4:**
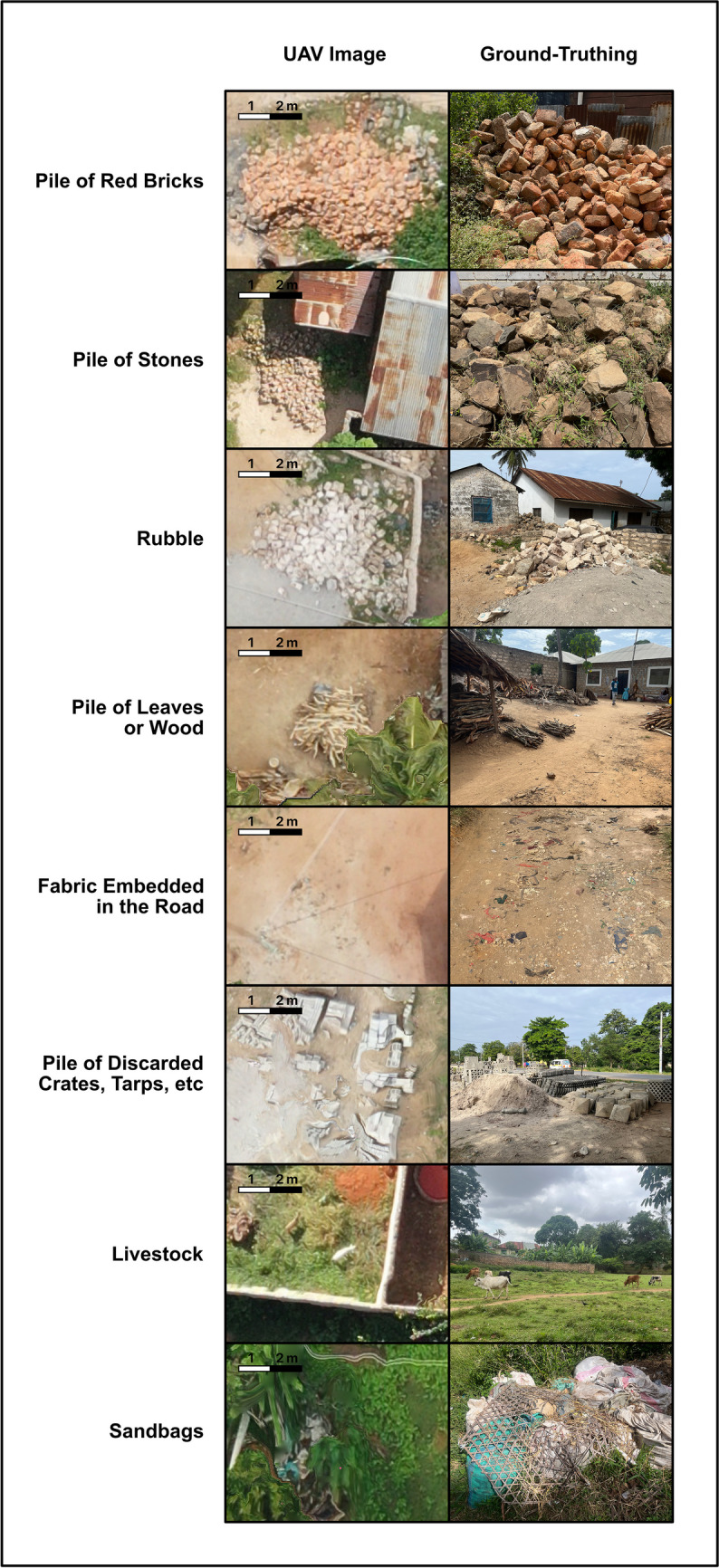
Trash mimics. Several items and non-trash piles could be mistaken for a trash pile on aerial imaging. Ground truthing was key for identifying these potential mimics so that they would not be classified as trash. Side by side comparisons are shown of the aerial image and ground truth image of each potential trash mimic

On aerial imaging, several items looked like trash, but key features help to distinguish them from dump sites (Figure 4). Piles of bricks, stones, or rubble create the texture of a trash pile but are typically more homogenous in color and could be predicted based on the site (white bricks in Kwale and red clay bricks in Kisumu). Piles of other items like leaves or wood also create a texture like a trash pile but have a more natural hue and the shapes of sticks were usually distinguished. Similarly, piles of discarded crates or tarps can usually be distinguished by their shapes and cannot typically hold sufficient rainwater for *Ae. aegypti* breeding. Fabric or even stones embedded in the road also create a texture and color similar to trash, but the location in the middle of the road helps to distinguish these features from piles of trash. A pile of white sandbags or a herd of cattle or goat could also initially look like white plastic bags, but the scale of these objects helps to prevent this misidentification.

### Unique trash types

In addition to the trash categories and mimics, there were special types of trash that were noted while walking through the communities which were not always readily distinguishable on aerial imaging and conferred a unique health risk. Several dump sites contained a large number of soiled diapers. Diapers do not hold water for *Ae. aegypti* to breed in, but they contaminate the local environment with feces, particularly during the rainy season; additionally, diapers naturally absorb large volumes of moisture and therefore are very difficult to burn. Another unique dump site consisted of a large area of crushed glass; again, this was not a mosquito risk but could pose an injury risk, particularly to anyone walking barefoot through the area. Finally, in both study areas, retired dump sites were identified where trash was no longer being dumped but the ground retained evidence of being a former dump. These former dump sites were difficult to distinguish from active dump sites on aerial imaging, demonstrating that despite trash removal, it can take years for these areas to be fully rehabilitated if ever.

## Discussion

Our study demonstrates that UAV imaging can be used to identify trash sites that serve as *Ae. aegypti* breeding grounds. Given the heterogeneity of trash dump sites, we created a trash classification scheme based on appearance on UAV imaging and risk level for serving as *Ae. aegypti* breeding habitat.

We created six categories of trash piles ranked based on overall *Ae. aegypti* risk which took into consideration a variety of factors, including pile density, surface area, ability to hold clean rainwater that would be feasible for *Ae. aegypti* oviposition and larval development, and likelihood of frequent disturbance of breeding sites. We also distinguished between high risk and no risk tires based on their visual appearance and ability to hold rainwater. This classification system codifies the expertise from our interdisciplinary team, including local expert entomologists, and the accumulated knowledge from numerous studies identifying trash and tires as common *Ae. aegypti* breeding sites (Hayes et al. 2003; Sekhon and Minhas 2014; Getachew et al. 2015; Ngugi et al. 2017; Mukhtar et al. 2018; Khan et al. 2023; Peña-García et al. 2023). We combined this knowledge of common breeding sites with a detailed review of over 3000 trash sites and nearly 1000 trash ground photos to create categories that could be used for aerial image classification of trash sites.

The definition and relevance of different trash types depends on the use case. Trash identification by UAV imaging is increasingly being deployed for various purposes, including mosquito abatement (Case et al. 2020; Schenkel et al. 2020; Valdez-Delgado et al. 2021; Lee et al. 2021), beach clean-up efforts (Andriolo et al. 2021; Liao and Juang 2022), and locating off-shore marine trash (Andriolo et al. 2022). In this study, we developed an aerial image classification system specific for trash that poses a risk for *Ae. aegypti* breeding. This classification incorporates subtle but meaningful differences in risk amongst trash and tire types based on distribution, location, and shape. For example, trash that is trampled, burned, submerged in dirty water, or tires that cannot hold water are considered differently than trash in large piles in relatively protected areas. Creating a classification system that accounts for these differences makes this a highly useful tool for studying the relationship between trash and diseases like dengue.

However, our study is limited to trash and tires and does not evaluate all potential *Ae. aegypti* breeding site such as open water containers, cisterns, or gutters, which have also been identified as possible breeding sites (Heukelbach et al. 2001; Mukhtar et al. 2018; Haddawy et al. 2019; Ngugi et al. 2020; Valdez-Delgado et al. 2021). Future studies carefully evaluating these sites with consideration of item use, temporal stability, disturbance, and water flushing effects could further expand the utility of UAV *Ae. aegypti* habitat mapping.

One important limitation of aerial imaging is that visualization of the ground can be obstructed by things overhead, such as overhanging eaves, trees, or dense vegetation. To address this limitation, we developed sub-categories which note trash that appears to be mixed with or partially obscured by trees or vegetation. While overhanging eaves can also limit views, this was encountered less frequently during the walk-throughs and tended to obscure only a small area of trash piles and therefore was not included in this sub-categorization. Additionally, unlike with ground visualization of a trash pile, the exact composition of a particular pile cannot be determined by these aerial images. However, during our walk throughs, it was noted that most community trash heaps consist of a mixture of different trash types, the vast majority of which included some types of containers that could serve as *Ae. aegypti* breeding sites; a few unique types of piles were noted but these were infrequent.

Our study is also limited in its evaluation of trash stability over time, including both turnover of trash within a pile and movement of individual piles over time. However, our assessment of trash disturbance took into consideration anecdotal observations by community leaders, stakeholders, and field staff about timing of trash turnover and stability of sites; in general, most of the larger and higher risk sites have reportedly been in place for many years with infrequent trash turnover; the smaller or sparser areas of trash are more difficult to assess but are suspected to be less stable. For particular cases such as burning, which causes frequent trash turnover, and trash dumped in a partially finished building, which may complete construction over a few months or years, we created sub-categories so that these changes could be accounted for or monitored over time. Repeated UAV flights and focused ground truth monitoring in the future would further enhance our understanding of trash turnover and dump site stability.

Trash is inherently difficult to classify, delineate boundaries around, and ultimately quantify. These challenges are shared by various methods of measuring trash in the environment, whether by aerial imaging (Schenkel et al. 2020; Andriolo et al. 2021; Lee et al. 2021), ground observations (Haddawy et al. 2019), or surveys (Getachew et al. 2015; Ngugi et al. 2017; Haddawy et al. 2019). However, aerial image analysis is a method that provides a map that can be re-referenced as classification systems evolve and other environmental variables are incorporated. These maps can provide quantifiable estimates of the surface area that trash is distributed within a given space and, by incorporating a classification system of risk and trash density, can give rough estimates of volume. As UAV trash assessments advance, additional research is needed to compare aerial image classification between different raters, against ground truth observations, during different seasons, and in other locations.

The classification scheme presented by this study serves as a foundation for future work using machine learning for automatic trash detection and assessment of *Ae. aegypti* breeding risk. Furthermore, the classification scheme creates different trash classes across a spectrum of *Ae. aegypti* risk and defines and justifies the visual features that impact the risk score. Even when focused specifically on trash dump sites, the surface area of the trash alone does not fully account for the quantity or quality of that trash as it pertains to *Ae. aegypti* risk. The classification scheme developed here paves the way for developing machine learning algorithms that factor in these nuanced but important differences. Additionally, identifying and quantifying the categories of trash according to the classification system developed creates an opportunity to measure the impact of interventions targeted at cleaning up and reducing high risk sites.

## Conclusions

Our aerial image classification system identifies trash, including discarded tires, across a range of *Ae. aegypti* breeding risk. Importantly, this study forms a relationship between the appearances of trash from UAV imaging and ground truth walk-throughs, and the corresponding risk of the trash site being a productive breeding ground. Existing studies have developed tools to quantify trash at the level of individual containers and larger environmental hotspots; this study adds to the rapidly expanding research using UAVs for trash identification by examining trash through the lens of *Ae. aegypti* breeding risk. This study highlights the varying risk of trash in different contexts depending on the density, area, water holding capacity, and level of disturbance of trash and tires. The development of this tool lays the foundation for further opportunities to use UAV imaging technology to efficiently and quantitatively evaluate environmental risk for *Aedes*-transmitted infectious diseases and to target and measure interventions aimed at mitigating that risk.

## Data Availability

Aerial images will be made available to researchers who provide a methodologically sound proposal. Proposals should be directed to jrosser@stanford.edu; to gain access, data requestors will need to sign a data access agreement.
